# On the spider genus *Arboricaria* with the description of a new species (Araneae, Gnaphosidae)

**DOI:** 10.3897/zookeys.558.6521

**Published:** 2016-02-02

**Authors:** Kirill G. Mikhailov

**Affiliations:** 1 Zoological Museum, Moscow Lomonosov State University, Bolshaya Nikitskaya Str. 6, Moscow 125009 Russia

**Keywords:** Spiders, Gnaphosidae, new species, taxonomy, Caucasus, Middle Asia

## Abstract

The spider genus *Arboricaria* Bosmans, 2000 is redefined and an updated diagnosis given. The differences between *Arboricaria* and *Micaria* Westring, 1851 are discussed in detail. A key to all five species of the genus is provided. One new species, *Arboricaria zonsteini* **sp. n.** (♂♀), is described based on specimens from Kyrgyzstan and Azerbaijan. One new synonym is proposed: *Arboricaria koeni* Bosmans in Bosmans & Blick, 2000, **syn. n.** is assigned to *Arboricaria sociabilis* Kulczyński in Chyzer & Kulczyński, 1897. Data on the distribution of *Arboricaria* in Russia and adjacent countries are presented with references to the papers on local spider faunas.

## Introduction

*Arboricaria* was established by Bosmans and Blick (2000) to accommodate the *Micaria subopaca* species group as outlined by Wunderlich (1980: 249). Five species were included, three of which had been known earlier, *Arboricaria cyrnea* (Brignoli, 1983) (the type species), *Arboricaria subopaca* (Westring, 1861) and *Arboricaria sociabilis* Kulczyński in Chyzer & Kulczyński, 1897, and two further described as new: *Arboricaria koeni* Bosmans in Bosmans & Blick, 2000 and *Arboricaria brignolii* Bosmans & Blick, 2000.

Platnick (2014, latest version), in his World Spider Catalog, does not accept this genus, because the authors provided “no evidence whatever that these taxa constitute the sister group of all other *Micaria*, or that the remaining *Micaria* do not constitute a paraphyletic group from which a relatively autapomorphic subgroup has been artificially extracted, those changes are not followed here”. The same concerns the current World Spider Catalogue (WSC 2015). *Arboricaria* is absent from the latest world gnaphosid revision as well (Murphy 2007), albeit it has never been synonymized with *Micaria*.

When preparing a review of the *Micaria* fauna of the former Soviet Union (Mikhailov 1987), I came across a specimen from Kyrgyzstan, Central Asia which showed a bifid male tibial apophysis and apparently represented a new species. Because its generic assignment seemed obscure at that time, this specimen was excluded from my 1987 paper. However, additional material has since become available from Azerbaijan, Caucasus.

The present contribution not only provides a description of that new species, but it also aims at clarifying the distinctions between two similar genera, *Micaria* Westring, 1851 and *Arboricaria* Bosmans, 2000, so as to provide a brief review of and a key to the known species of the latter genus. In addition to Mikhailov’s (1987) faunistic review, data on the distribution of *Arboricaria* species in Russia and adjacent countries are provided. Since most of the species included in *Arboricaria* are well-known and properly described, e.g. by Wunderlich (1980) within *Micaria* and/or by Bosmans and Blick (2000) in *Arboricaria*, this paper requires no redescriptions to be made and can be reduced to a key, with only short remarks given for most of species.

## Material and methods

Material of three species was examined in detail: *Arboricaria subopaca*, *Arboricaria sociabilis* and *Arboricaria zonsteini* sp. n. Specimens were examined using MBS-9 and Olympus stereo microscopes. All initial pencil sketches drawn on scale paper were subsequently inked and then digitized with Cintiq.

The following abbreviations are used below: ap – apically, Cb – cymbium, d – dorsally, F – femur, Mt – metatarsus, pl – prolaterally, Pt – patella, T – tarsus, Ti – tibia, IRSNB – Institut Royal des Sciences Naturelles de Belgique, Bruxelles, ZMMU – Zoological Museum, Moscow State University, Russia. All measurements are given in mm.

Only basic and necessary synonymies are given in the species reviews below, as a more detailed list is available in WSC (2015).

Data on the distribution of *Arboricaria* species in Russia and Azerbaijan are mostly previously unpublished (my unpublished card Catalogue of the Spiders of Russia and Adjacent Territories; see also Mikhailov 2012, 2013). Only well-figured descriptions and redescriptions as well as main synonyms are listed here.

## Taxonomy

### Arboricaria

Taxon classificationAnimaliaAraneaeGnaphosidae

Bosmans, 2000

Arboricaria Bosmans, in Bosmans and Blick 2000: 460–461.
Arboricaria Tuneva 2007: 250.

#### Type species.

*Micaria cyrnea* Brignoli, 1983.

#### Composition.

*Arboricaria* includes five known species listed above and one new species described below.

Despite not being followed on the world spider catalogues (see above), the original description of *Arboricaria* and its diagnosis both fully fit the provisions of the International Code of Zoological Nomenclature, especially Articles 13.1 and 67.4 (ICZN 1999), i.e., diagnostic characters are sufficient for recognizing the new genus, as well as the type species is properly indicated. So there are no formal grounds to reject the validity of *Arboricaria*.

According to the original diagnosis, the new genus “is very close to *Micaria* and differs by the more flattened, wider cephalothorax, the less spinate legs and the posteriorly truncate sternum. Males differ by the large tibial apophysis, bifid or curved, the bulging bulbus and the absence of the median apophysis (= Retinaculum in Wunderlich 1980), females by the large epigyneal fossa [= groove] with distinctly chitinized posterior margin”. In addition, the *Micaria subopaca*-group is characterized by 0-2 distal-ventral spines on the cymbium, as well as the absence of ventral spines on tibiae and metatarsi I–II (Wunderlich 1980: 249).

Not all of the characters are equally important.

The width of the carapace is variable within the remaining *Micaria* (cf. Table 1 herein with table 1 in Wunderlich 1980). In *Micaria* sensu stricto, the carapace length/width index is 1.2–2.0.

**Table 1.** Carapace length/width index in *Arboricaria* species.

Species/Sex	Index	Source
*Arboricaria zonsteini* sp. n., ♂	1.29–1.31	Present paper
*Arboricaria zonsteini* sp. n., ♀	1.4	Present paper
*Arboricaria brignolii* Bosmans & Blick, 2000, ♂	1.32–1.33	Bosmans and Blick 2000
*Arboricaria brignolii* Bosmans & Blick, 2000, ♀	1.35, 1.46	Bosmans and Blick 2000
*Arboricaria koeni* Bosmans in Bosmans & Blick, 2000, ♂	1.33–1.37	Bosmans and Blick 2000
*Arboricaria koeni* Bosmans in Bosmans & Blick, 2000, ♀	1.42	Bosmans and Blick 2000
*Arboricaria cyrnea* (Brignoli, 1983), ♂	1.35–1.36	Bosmans and Blick 2000
*Arboricaria cyrnea* (Brignoli, 1983), ♀	1.47	Bosmans and Blick 2000
*Arboricaria subopaca* (Westring, 1861), ♂,♀	1.25–1.35	Wunderlich 1980

The same concerns the size of the tibial apophysis (for large ones in *Micaria*, see figs 29a, 31a, in Wunderlich 1980), not bifid in *Micaria*, as well as in *Arboricaria subopaca*.

A median apophysis is absent or almost absent in *Micaria rossica* Thorell, 1875, wholly absent both in *Micaria utahna* Gertsch, 1933 and *Micaria medica* Platnick & Shadab, 1988.

An analysis of leg spination (see table 1 in Wunderlich 1980: 250–251) shows that *Arboricaria* species fall within the range of *Micaria* variability, yet close to its marginal part.

**Figures 1–5.**
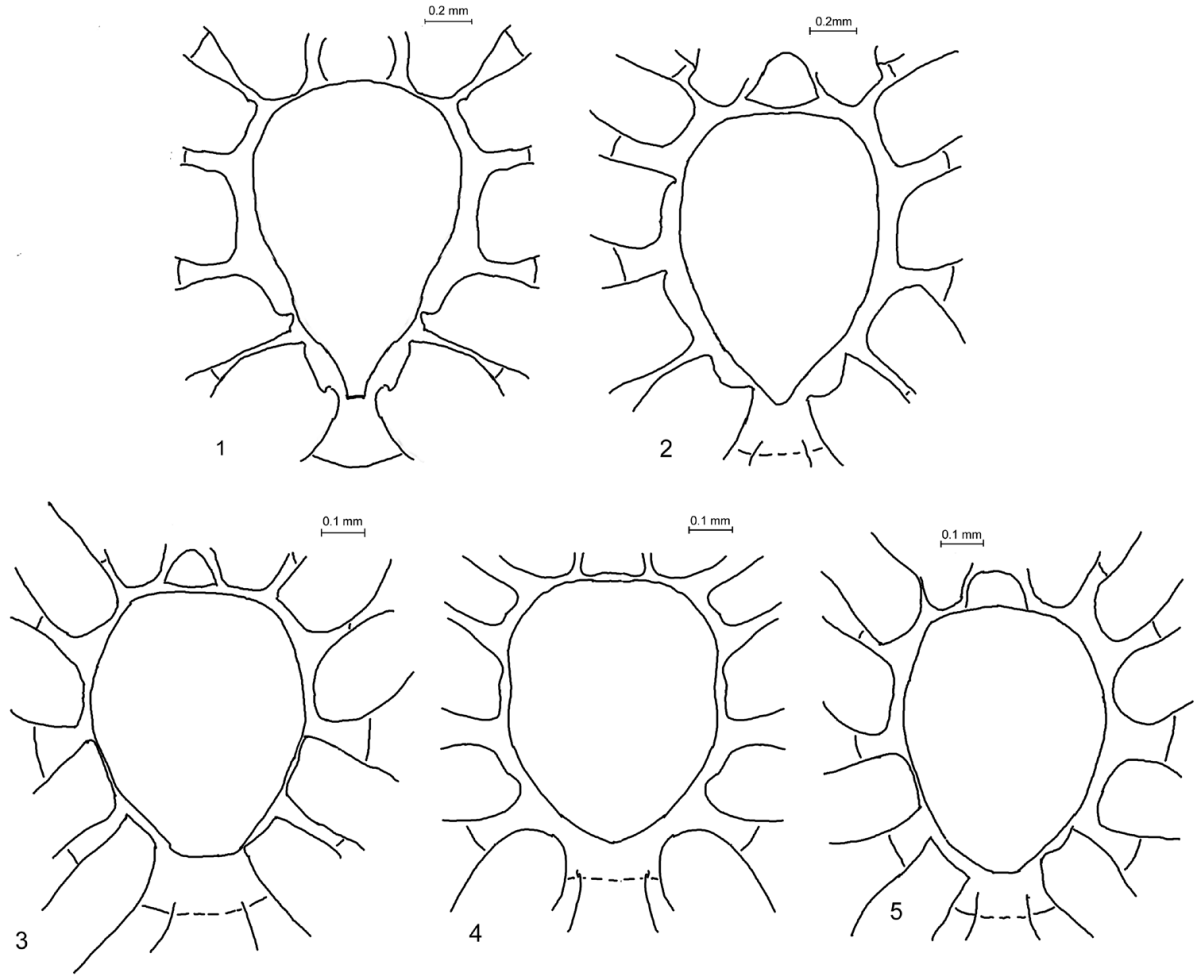
Sternum in *Micaria* and *Arboricaria*. **1** *Micaria formicaria*, male **2** *Micaria fulgens*, female **3** *Arboricaria subopaca*, female **4** *Arboricaria zonsteini* sp. n., male **5** *Arboricaria zonsteini* sp. n., female.

The shape of the posterior part of the sternum is clearly different in *Micaria* and *Arboricaria* (see Figs 1–5).

Therefore, the above diagnosis of *Arboricaria* must be adjusted. This genus is indeed close to *Micaria*, but differs in the following characters that together allow recognizing the genus: a posteriorly truncate sternum in both sexes, a bulging bulbus and a missing median apophysis, a chiefly bifid tibial apophysis, a large epigynal groove with distinctly chitinized posterior margins in females. At least, the shape of bulbus and bifid apophysis can be regarded as apomorphic characters. All these characters constitute *Arboricaria* as a monophyletic and sister-group to other *Micaria*. An extended description of *Arboricaria* is available in Bosmans and Blick (2000).

The distribution pattern of *Arboricaria* is mostly Mediterranean and on the mountain regions of central Asia, although *Arboricaria subopaca* extends to most of the Palearctic.

### Micaria

Taxon classificationAnimaliaAraneaeGnaphosidae

Westring, 1851

#### Remarks.

Type species. *Micaria fulgens* (Walckenaer, 1802), originally described as *Aranea fulgens*.

#### Diagnosis.

Gnaphosids of the “*Micaria*-group” (Murphy 2007), differing from *Arboricaria* by the more or less ovoid, posteriorly not truncate sternum in both sexes, the ovoid, not bulging bulbus with a mostly present median apophysis, the palpal tibial apophysis, sometimes poorly expressed, not bifid in males, the epigynal groove in females, if present, without distinctly chitinized posterior margins.

#### Composition.

101 species (WSC 2015).

#### Distribution.

Holarctic. Other records require confirmation.

An analysis of the new *Micaria* species described from the Palaearctic since Bosmans and Blick (2000), all listed in WSC (2015), shows no match with *Arboricaria* characters. Therefore, despite the previous neglect of *Arboricaria*, no new species of this genus have been described within *Micaria* *sensu lato* since 2000. In addition, all extra-Holarctic records of *Micaria* are doubtful; these species most likely belonging to other genera or even families (Murphy 2007).

### Key to *Arboricaria* species

**Table:** 

1	Males	**2**
–	Females	**6**
2	Tibial apophysis not bifurcate (see fig. 35b in Wunderlich 1980); complex of other male diagnostic characters of *Arboricaria* present	***Arboricaria subopaca***
–	Tibial apophysis bifurcate	**3**
3	Branches of tibial apophysis of equal or subequal length	**4**
–	Branches of tibial apophysis different in length	**5**
4	Embolus wide and large, rising over bulbus (Figs 17–20)	***Arboricaria sociabilis***
–	Embolus thin, lying directly on apical surface of bulbus (Fig. 6)	***Arboricaria zonsteini* sp. n.**
5	Inner branch of tibial apophysis ca 3 times longer than outer branch; maximum width of embolus closer to 1/4 of bulbus width (see figs 24 & 25 in Bosmans and Blick 2000)	***Arboricaria cyrnea***
–	Inner branch of tibial apophysis ca 2 times as long as outer branch; maximum width of embolus closer to 1/2 of bulbus width (see figs 28 & 29 in Bosmans and Blick 2000)	***Arboricaria brignolii***
6	Lateral edges of epigynal groove divergent (see fig. 59 in Wunderlich 1980)	***Arboricaria subopaca***
–	Lateral edges of epigynal groove parallel or convergent	**7**
7	Lateral edges of epigynal groove parallel (see fig. 30 in Bosmans and Blick 2000)	***Arboricaria brignolii***
–	Lateral edges of epigynal groove convergent	**8**
8	Spermathecae shorter than epigynal groove; spermathecae not reaching the latter’s fore edge (Figs 10–11)	***Arboricaria zonsteini*** sp. n.
–	Spermathecae long, reaching fore edge of epigynal groove or even exceeding it	**9**
9	Hind edge of epigynal groove straight (see fig. 26 in Bosmans and Blick 2000)	***Arboricaria cyrnea***
–	Hind edge of epigynal groove protruding backwards (see fig. 60a in Wunderlich 1980 and fig. 34 in Bosmans and Blick 2000; fig. 60b–c in Wunderlich 1980 refers to *Arboricaria brignolii*, see below)	***Arboricaria sociabilis***

### Description

#### Arboricaria zonsteini sp. n.

Taxon classificationAnimaliaAraneaeGnaphosidae

http://zoobank.org/D362E1C2-E41A-4AAF-AECA-C4F3FAFC6CA0

**Figures 6–9.**
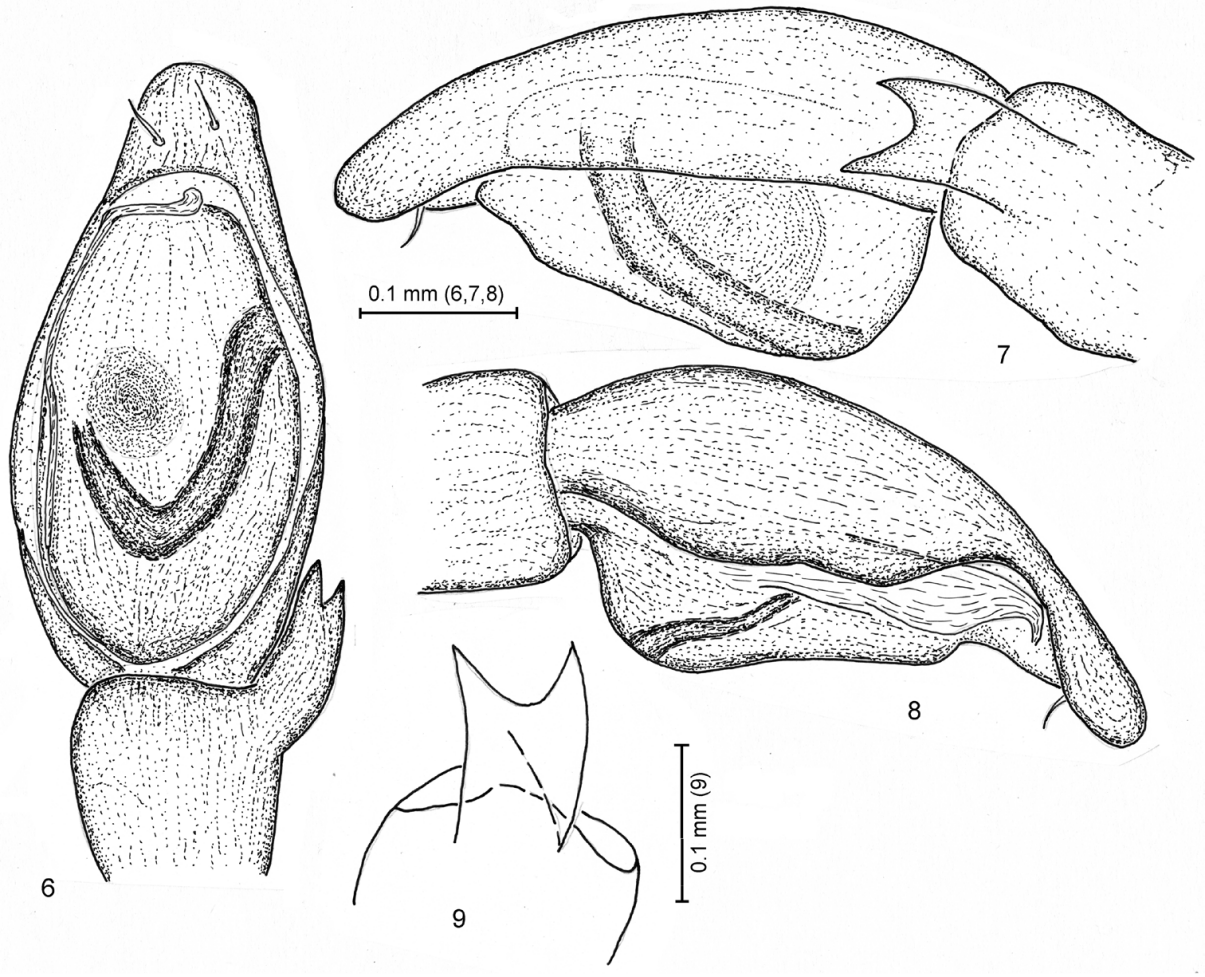
*Arboricaria zonsteini* sp. n., right male palp. **6** ventral view **7** retrolateral view **8** prolateral view **9** tibial apophysis, schematically.

**Figures 10–11.**
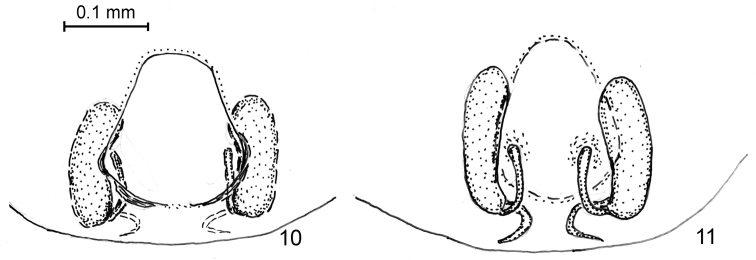
*Arboricaria zonsteini* sp. n., female copulatory organs. **10** epigyne **11** vulva.

Figs 5 , 6–9 , 10–11

##### Material.

Holotype ♂ (ZMMU Ta-7739), Kyrghyzstan, env. of Frunze (now Bishkek), ca 42°52-54'N, 74°33-40'E, 30.03.1983 (S.L. Zonstein & S.V. Ovtchinnikov). Paratypes, Azerbaijan, Apsheron Peninsula: 1 ♂ (ZMMU Ta-7740), Baku City, environs of Lake Ganly-Gyol, ca 40°22'N, 49°48'E, shrub branch, 21.05.1996 (E. Huseynov); 1 ♀ (ZMMU Ta-7741), Baku City, Botanical Garden, 40°21'20"N, 49°49'46"E, pine trunk, 13.06.1996 (E. Huseynov); 1 ♀ (ZMMU Ta-7742), Mardakyany, 40°29'32"N, 50°08'20"E, stone wall, 1.06.1996 (E. Huseynov).

##### Name.

Honours Sergei L. Zonstein, arachnologist, now living in Israel, earlier in Kirghizia (= Kyrgyzstan).

##### Diagnosis.

The new species differs by a combination of the following characters: Males: equally long branches of tibial apophysis with thin embolus lying on apical surface of bulbus; Females: convergent edges of epigynal groove with moderately long spermathecae, the latter being shorter than the groove, the former not reaching the fore edge of the latter.

##### Description.

**Male** (holotype; measurements of paratype in brackets). Carapace length 1.20(1.05), width 0.93(0.80), ratio 1.29(1.31). Carapace and leg femora reddish brown, in holotype carapace darker, other podomeres, especially metatarsi and tarsi, straw-coloured.

For leg measurements, see Table 2.

**Table 2.** Leg measurements of male *Arboricaria zonsteini* sp. n.

Leg/Article	F	Pt	Ti	Mt	T
I	0.79(0.75)	0.46(0.40)	0.69(0.58)	0.57(0.45)	0.50(0.40)
II	0.79(0.73)	0.41(0.40)	0.63(0.55)	0.56(0.45)	0.51(0.40)
III	0.64(0.60)	0.36(0.30)	0.49(0.40)	0.53(0.40)	0.37(0.30)
IV	0.81(0.78)	0.43(0.38)	0.79(0.65)	0.79(0.58)	0.43(0.40)
Total	3.03(2.86)	1.66(1.48)	2.60(2.18)	2.45(1.88)	1.81(1.50)

Leg spination: F I d 1, pl 1, F II–IV d 1, Ti III–IV pl 1(ap), Mt III v 2(1.2, 2.2), Mt IV v 1.1.1.2 (1.1.2).

Abdomen length 1.63(1.60), width 0.93(0.93), ratio 1.76(1.72), dark brown, with transverse band of white bristles, broken in the middle.

Palpus as in Figs 6–9. Length of palpomeres (holotype): F 0.37, Pt 0.21, Ti 0.16, Cb 0.50. Cymbium longer than femur, rounded in apical part, with 2 ventral-distal spines. Tibial apophysis long, reaching ca ½ of tibia length, wide, with parallel margins, bifid, with acute apices. Cymbium apical part shorter than tibial apophysis. Tegulum oval in plane, without conical apophyses. Embolus poorly chitinized, lying directly on apical surface of tegulum. Subtegulum not visible.

**Female.** Carapace length 1.05, 1.05, width 0.75, 0.75, ratio 1.4, 1.4. Body coloration as in male, but carapace being pale reddish brown. For leg measurements, see Table 3 (in all female measurements, the first one is for the paratype from Mardakyan, the second for that from the Botanical Garden).

**Table 3.** Leg measurements of female *Arboricaria zonsteini* sp. n.

Leg/Article	F	Pt	Ti	Mt	T
I	0.63, 0.70	0.35, 0.38	0.48, 0.55	0.40, 0.45	0.38, 0.43
II	0.60, 0.68	0.30, 0.38	0.48, 0.55	0.40, 0.45	0.38, 0.38
III	0.53, 0.58	0.30, 0.28	0.38, 0.43	0.38, 0.43	0.30, 0.40
IV	0.75, 0.75	0.33, 0.35	0.65, 0.68	0.60, 0.68	0.38, 0.40
Total	2.51, 2.71	1.28, 1.39	1.99, 2.21	1.78, 2.01	1.44, 1.61

Leg spination: F I d 1, pl 1, F II–IV d 1, Ti III–IV pl 1(ap), Mt III–IV v 1.2.

Abdomen length 1.55, 1.88, width 0.78, 1.00, ratio 1.88, 1.99.

Epigyne and vulva as in Figs 10–11. Epigynal groove subpyriform, as long as wide, with slightly convex edges; distance between its posterior edge and epigastric furrow being ¼ of groove length. Copulatory openings small (like in most *Arboricaria* and *Micaria*), lying at lateral edges of groove in its posterior one-third. Copulatory tubes thin, almost vertical and parallel to each other, about half the length of spermathecae. Spermathecae oblong-oval, parallel to each other, being 2/3–3/4 as long as epigynal groove.

##### Distribution.

Northern Kyrgyzstan and Apsheron Peninsula (Azerbaijan).

#### Arboricaria cyrnea

Taxon classificationAnimaliaAraneaeGnaphosidae

(Brignoli, 1983)

Micaria canestrinii : Wunderlich 1980: 292–293, figs 37a–d (♂).
Micaria cyrnea Brignoli, 1983: 564 (*nomen novum*).
Arboricaria cyrnea : Bosman and Blick 2000: 461–463, figs 24–31 (♂♀).
Arboricaria cyrnea Note. This is the type species of the genus.

##### Distribution

(after Bosmans and Blick 2000). Greece: north Aegean Islands; Italy (continental); France: Corsica. The records from Russia are erroneous (see below under *Arboricaria sociabilis*).

##### Remark.

The new name was proposed by Brignoli (1983) for *Micaria canestrinii* Roewer, 1951 as misidentified by Wunderlich (1980).

#### Arboricaria subopaca

Taxon classificationAnimaliaAraneaeGnaphosidae

(Westring, 1861)

Fig. 3

Micaria subopaca Westring, 1861: 336.
= Micaria albostriata L. Koch, 1877.
= Micaria humilis Kulczyński, 1885.
Micaria subopaca : Wunderlich 1980: 290–291, figs 35a–e, 59 (♂♀).

##### Material.

1 ♂, 1 ♀ (ZMMU Ta-2119), Russia, Moscow Area, environs of Bolshevo, 55°56'N, 37°51'E, under pine bark, 28.02.1926 (leg. et det. V.I. Pereleshina); 1 ♀ (ZMMU), Belarus, Minsk Area, Myadel Distr., Lake Naroch, ca 54°49-53'N, 26°40-50'E, song thrush nest, 11.07.1967 (leg. A.S. Gembitsky, det. E.M. Zhukovets); 1 ♀ (ZMMU), Belarus, Minsk Area, Soligorsk Distr., Velichkovichi, ca 52°37'N, 27°14-15'E, 12.05.1982 (leg. Yu.M. Zhukovets, det. K.G. Mikhailov).

##### Distribution.

All Europe north to Norway. Russia east to Urals, with scattered records in Transbaikalia and Kamchatka.

##### Russia and adjacent countries

(all as *Micaria*, exceptions are marked).

Russia. Karelia (Palmgren 1943). Leningrad Area (Charitonov 1928, as *Micaria albostriata*; Oliger 2010). Moscow Area (Pereleshina 1928, as *Micaria albostriata*; Mikhailov 1987). Ryazan Area (Mikhailov 1987). Kaluga Area (Esyunin et al. 1993). Lipetsk Area (Panteleeva 1982, as *Micaria albostriata*). Voronezh Area (Panteleeva 2007). Belgorod Area (Kulczyński 1913, as *Micaria albostriata*; Ponomarev and Polchaninova 2006, as *Arboricaria*). Ulyanovsk Area (Kuzmin and Alekseenko 2011). Samara Area (Krasnobaev and Ovtsharenko 1986; Krasnobaev and Matveev 1993; Krasnobaev 2004, 2007). Volgograd Area (Ponomarev and Khnykin 2013, as *Arboricaria*). Rostov Area (Ponomarev et al. 2006; Ponomarev and Lebedeva 2014, both as *Arboricaria*). Komi Republic: Pechoro-Ilychskiy Nature Reserve (Kazantsev 2013, as *Arboricaria*). Sverdlovsk Area (Tuneva 2007, as *Arboricaria*). Perm Area (Esyunin and Efimik 1995; Tuneva 2007, as *Arboricaria*). Chelyabinsk Area (Esyunin and Efimik 1996; Tuneva 2007, as *Arboricaria*). SE part of West Siberia (Romanenko 2007). Altai Province (Azarkina and Trilikauskas 2013). Krasnoyarsk Province: Stolby Nature Reserve (Tuneva 2007, as *Arboricaria*). Buryatia (Danilov 1993, 2008). Kamchatka (Kulczyński 1885, as *Micaria humilis*; 1926, as *Micaria albostriata*).

Estonia (Vilbaste 1987).

Lithuania (Pupiska 1939, as *Micaria albostriata*; Vilkas 1992; Biteniekytė and Rėlys 2011).

Latvia (Šternbergs 1981, as *Micaria albostriata*).

Belarus: Minsk Area (Gembitsky et al. 1985).

Ukraine. Chernovtsy Area (Fedoriak and Rudenko 2007). Chernigov Area (Evtushenko 1992). Lugansk Area (Polchaninova and Prokopenko 2013). Donetsk Area (Polchaninova and Prokopenko 2008). Kherson Area (Talanov and Nazarenko 1989) [doubtful data; confirmation needed (Polchaninova and Prokopenko 2013)].

Moldova (Roşca 1941, as *Micaria albostriata*).

#### Arboricaria sociabilis

Taxon classificationAnimaliaAraneaeGnaphosidae

Kulczyński, 1897

Figs 12–16 , 17–20

Micaria sociabilis Kulczyński in Chyzer & Kulczyński, 1897: 254 & 255 (key), 258–259, Tab.X., figs 21 (♀) 25a–b (♂).
Micaria canestrinii Roewer, 1951: 447 (replacement name for *Micaria aurata* Canestrini, 1868, praeocc.).
Micaria sociabilis : Wunderlich 1980: 291–292, figs 36a–b (♂, doubtful, see note in the text below, incorrect drawings), 60a (♀).
Arboricaria koeni Bosmans in Bosmans & Blick, 2000: 465, figs 32–35 (♂♀), **syn. n.**
Micaria sociabilis : Pfliegler 2014: 145 (record), figs 3e, 4c (♂♀).
Micaria sociabilis : Sentenská et al. 2015: figs 4, 6 (♂♀).
NotMicaria sociabilis : Wunderlich 1980: figs 60b–c (♀, = *Arboricaria brignolii*, see below).

##### Material.

1 ♂ (IRSNB, holotype of *Arboricaria koeni*), Greece, Kreta, Chania, [in bark], 22.V.1994 (leg. Koen van Keer); 1 ♂ (IRSNB, paratype of *Arboricaria koeni*, left palp missing), Kreta, Chania, 22-5-1994; 1 ♂ (ZMMU; left palp and leg I only), Russia, Rostov-on-Don, 47°13'33"N, 39°41'59"E, window of living flat, 8.06.1978 (leg. et det. A.V. Ponomarev).

##### Taxonomic remarks.

Originally, the male was matched with the female with some doubts (Chyzer and Kulczyński 1897), because they were taken from different, but not extremely distant localities of the former Austro-Hungarian Empire. Syntypes (1 ♂, 1 ♀, “Ungarn” = “Hungary”) are listed by Wunderlich (1980), but he only redescribed the female. Comparing the epigynes of *Arboricaria sociabilis* and *Arboricaria koeni* shows no essential difference between them; therefore, these names are to be synonymized. The position of the copulatory openings is a little variable; in the type of *Arboricaria sociabilis*, they are closer to the middle part of the epigynal groove, in the *Arboricaria koeni* type and the *Arboricaria sociabilis* material as depicted by Pfliegler (2014) closer to the posterior one-third.

**Figures 12–16.**
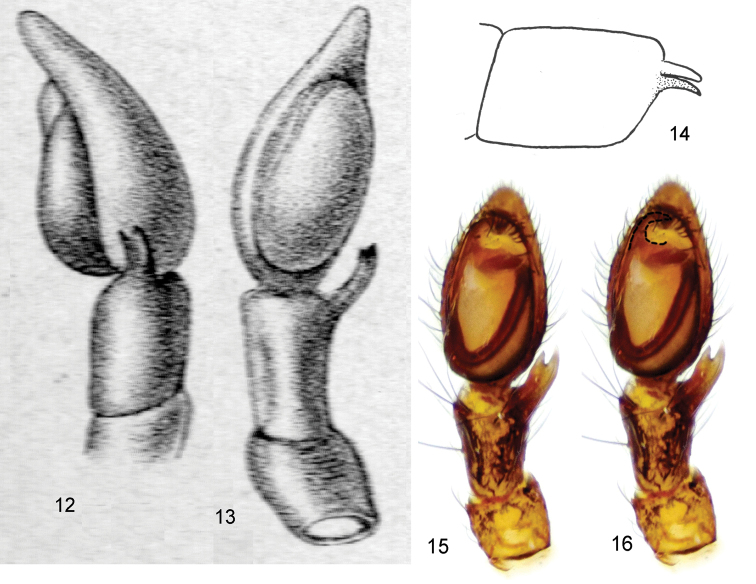
*Arboricaria sociabilis*, male palp, from different sources. **12**, **13** original drawings by Chyzer and Kulczynski (1897) **14** “improved” drawing by Wunderlich (1980) **15** original photo from Pfliegler (2014) paper **16** same photo with traced embolus. No scale. 12, 13, no copyright, 14, with permission of Joerg Wunderlich, 15, courtesy of W. Pfliegler (Debrecen, Hungary).

A male syntype of *Micaria sociabilis* from Mukachevo is currently kept in the Zoological Museum in Warsaw, Poland, but both palps are missing (W. Wawer, pers. comm.). The tibial apophysis as redrawn by Wunderlich (1980: Fig. 36a, see also Fig. 14) from the original description (Chyzer and Kulczyński 1897: tab. X, fig. 25b, see also Fig. 12) is certainly incorrect. No deep bifurcation is visible in the original figure. Miller (1971) in his key to Czechoslovak spiders, pointed out: “Tibial apophysis apically [sic! – *KM*] forked with 2 teeth, lower tooth narrower and more pointed; it is laterally slightly bent, ventrally rounded outside and bent forward and it has the same width” (translated from Czech by A. Šestaková). Miller’s specimen of *Micaria sociabilis* was never depicted and is currently missing among the other *Micaria* samples kept in the National Museum in Prague, Czech Republic (Kůrka 1994).

**Figures 17–20.**
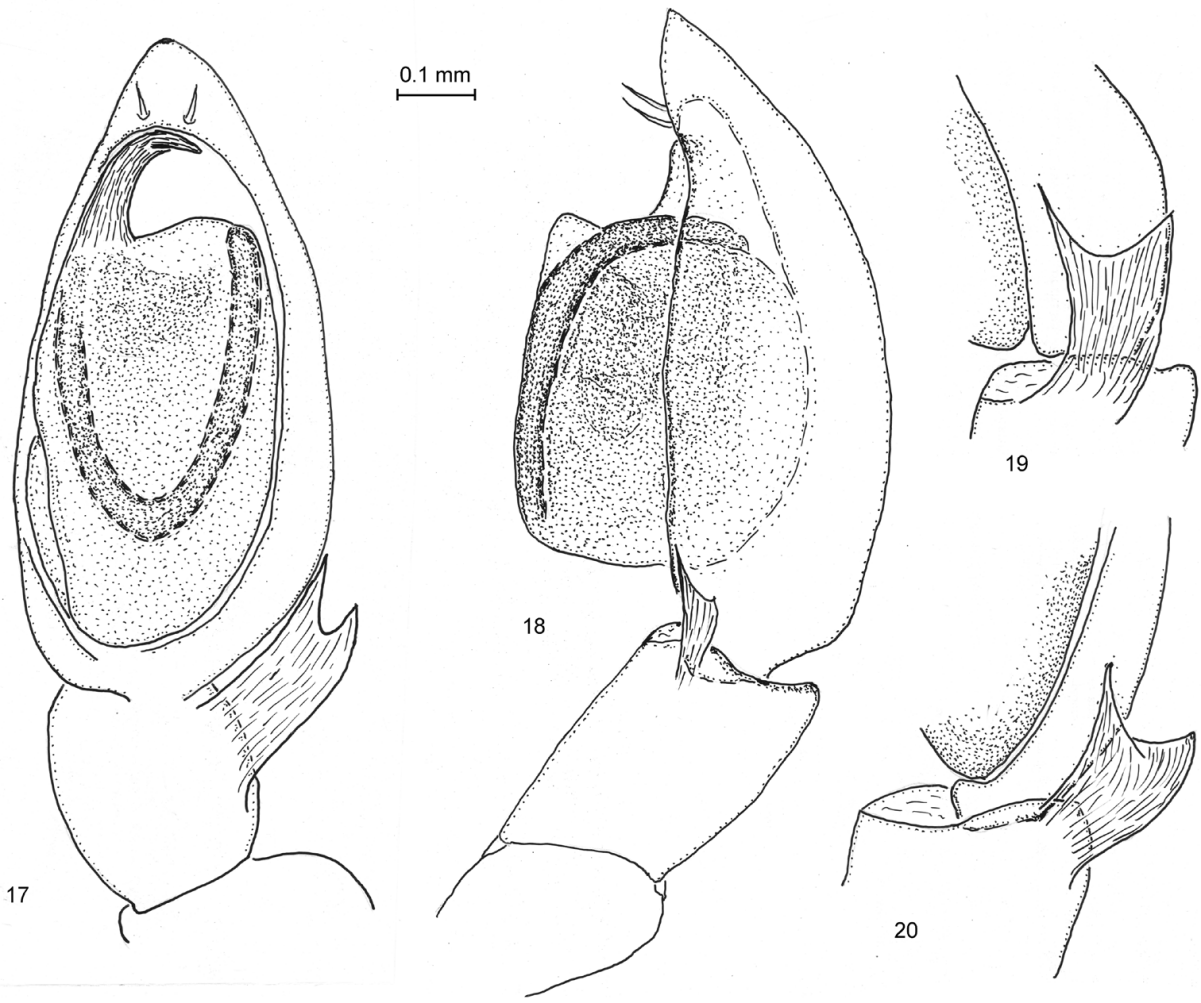
*Arboricaria sociabilis*, male palp from ZMMU collection. **17** ventral view **18** retrolateral view **19**, **20** tibial apophysis, different projections.

**Figure 21.**
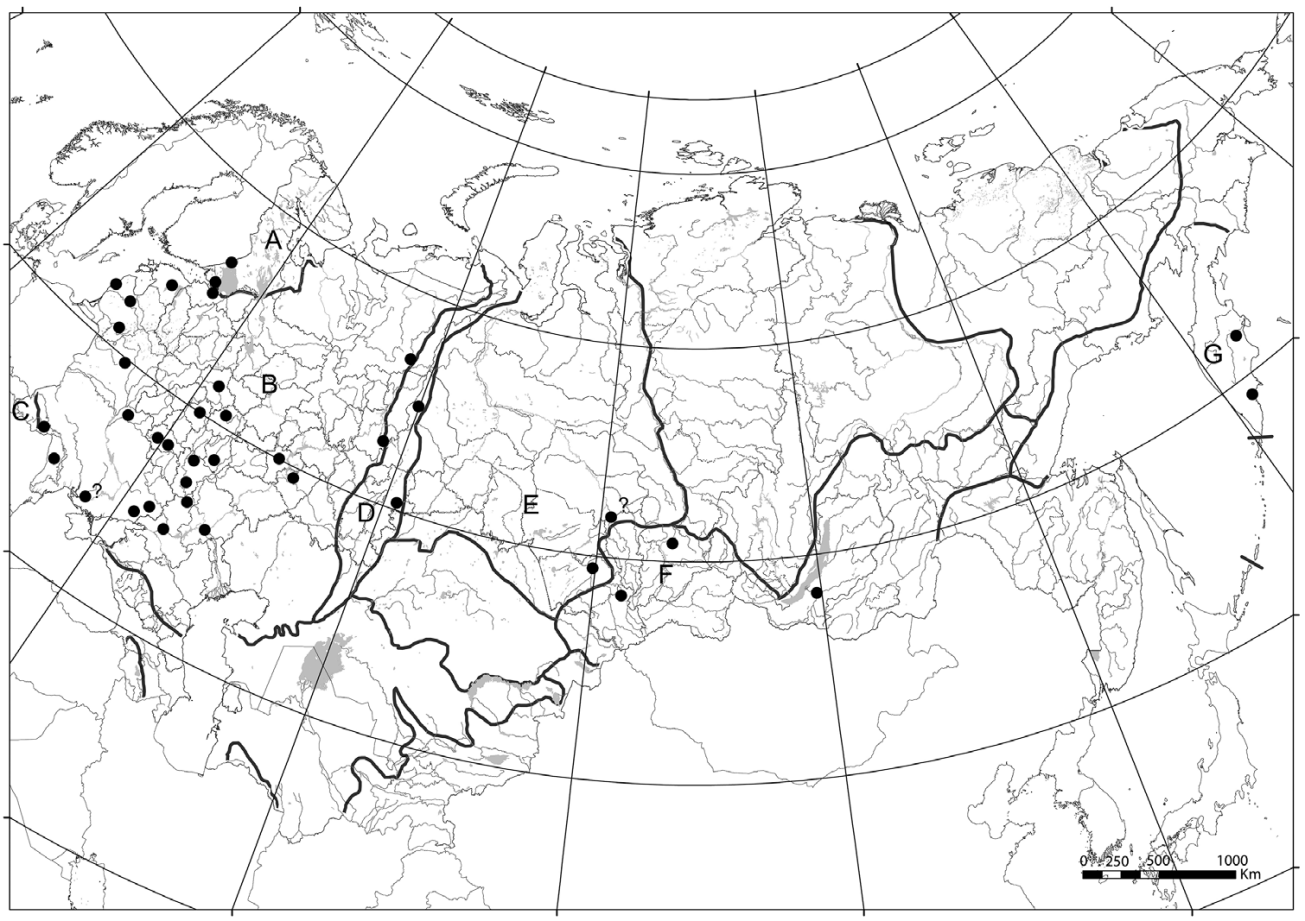
Localities of *Arboricaria subopaca* in Russia and other post-Soviet republics. Main physiographical areas accepted in Mikhailov (1997, 2013) are indicated: **A** Fennoscandia **B** Russian Plain **C** Carpathians **D** Urals **E** West Siberia **F** mountains of South Siberia **G** Kamchatka.

The picture of the *Micaria sociabilis* male palp as presented by Pfliegler (2014) certainly indicates the identity of this species with *Arboricaria koeni* (male types examined, see above in Material) and additionally confirms the synonymy of these names. To be exact, not enough details are visible in the publication, but a correct shape of the embolus is shown in the original photograph kindly sent to me by the author (cf. Figs 15 and 16 with traced embolus).

Characteristically, a male specimen from Rostov-on-Don was initially identified by A.V. Ponomarev as *Micaria sociabilis*, only later re-labeled as *Arboricaria koeni*.

##### Distribution.

Ukraine: Transcarpathia: Mukachevo (= Munkácz in Chyzer & Kulczyński [1897]). NE-Hungary (two other localities from the original description; Debrecen [Pfliegler 2014]); Spain, continental France, together with Corsica, Italy, Croatia, Macedonia, continental Greece, together with Crete, Bulgaria, Romania, Czech Republic, Slovakia (Helsdingen 2014, for *Micaria sociabilis* and *Micaria koeni*). Russia: Rostov Area (Ponomarev and Tsvetkov 2006a, as *Arboricaria cyrnea*; Ponomarev 2008; Ponomarev and Dvadnenko 2013), Krasnodar Province: Kushchevskaya (Ponomarev and Tsvetkov 2006b, as *Arboricaria brignolii*; Ponomarev 2008). Azerbaijan (Caucasus Major: Huseynov, Alieva, 2010, as *Arboricaria koeni*, Apsheron Peninsula: Huseynov 2002, as *Arboricaria koeni*), all for *Micaria* (or *Arboricaria*) *koeni*. The records of *Arboricaria cyrnea* from the Rostov Area, as well as those of *Arboricaria brignolii* from the Rostov Area and Krasnodar Province belong to *Arboricaria sociabilis* (A.V. Ponomarev, pers. comm., as *Arboricaria koeni*).

##### Biology.

See Sentenská and Pekár (2013), Sentenská et al. (2015).

#### Arboricaria brignolii

Taxon classificationAnimaliaAraneaeGnaphosidae

Bosmans & Blick, 2000

Micaria ? sociabilis : Wunderlich, 1980: Fig. 60b, c.
Arboricaria brignolii Bosmans & Blick, 2000: 463–465, figs 28–31 (♂♀).

##### Distribution

(after Bosmans and Blick 2000). Portugal, France: Dept. Var: Le Lavandon (new). Records from Russia are erroneous (see above, under *Arboricaria sociabilis*).

##### Remark.

As it was already pointed out by Bosmans and Blick (2000), with some doubts, the record of *Micaria sociabilis* from France by Wunderlich (1980: 291) is referred to *Arboricaria brignolii*. I support this reference.

## Supplementary Material

XML Treatment for Arboricaria

XML Treatment for Micaria

XML Treatment for Arboricaria zonsteini

XML Treatment for Arboricaria cyrnea

XML Treatment for Arboricaria subopaca

XML Treatment for Arboricaria sociabilis

XML Treatment for Arboricaria brignolii
